# Supplementary material for: Are food and drink retailers within NHS venues adhering to NICE Quality standard 94 guidance on childhood obesity? A cross-sectional study of two large secondary care NHS hospitals in England
Source: BMJ Open. 2017 Nov 16;7(11):e018214. doi: 10.1136/bmjopen-2017-018214 (PMC5701976; doi:10.1136/bmjopen-2017-018214)
Supplement: Author's manuscript [file bmjopen-2017-018214.draft_revisions.pdf]

BMJ Open is committed to open peer review. As part of this commitment we make the peer review history of every article we publish publicly available.

When an article is published we post the peer reviewers' comments and the authors' responses online. We also post the versions of the paper that were used during peer review. These are the versions that the peer review comments apply to.

The versions of the paper that follow are the versions that were submitted during the peer review process. They are not the versions of record or the final published versions. They should not be cited or distributed as the published version of this manuscript.

BMJ Open is an open access journal and the full, final, typeset and author-corrected version of record of the manuscript is available on our site with no access controls, subscription charges or pay-per-view fees (http://bmjopen.bmj.com).

If you have any questions on BMJ Open's open peer review process please email editorial.bmjopen@bmj.com

BMJ Open

# Are food and drink retailers within NHS venues adhering to NICE Quality Standard 94 guidance on childhood obesity?

| | |
|---|---|
| Journal: | *BMJ Open* |
| Manuscript ID | bmjopen-2017-018214 |
| Article Type: | Research |
| Date Submitted by the Author: | 14-Jun-2017 |
| Complete List of Authors: | James, Alice ; University of Bristol, 5th year Medical Student, Faculty of Health Sciences<br>Birch, Laura; University of Bristol, NIHR Biomedical Research Centre Bristol (Nutrition Theme)<br>Fletcher, Peter; Cheltenham General Hospital, Department of General and Old Age Medicine<br>Pearson, Sally; Gloucestershire Hospitals NHS Foundation Trust Main Board, Department of Clinical Strategy<br>Boyce, Catherine; Gloucestershire Hospitals NHS Foundation Trust, Department of Clinical Strategy<br>Ness, Andy; University of Bristol, NIHR Biomedical Research Centre Bristol (Nutrition Theme)<br>Shield, Julian; University of Bristol, NIHR Biomedical Research Centre (Nutrition Theme)<br>Lithander, Fiona; University of Bristol, NIHR Biomedical Research Centre (Nutrition Theme) |
| <b>Primary Subject Heading</b>: | Paediatrics |
| Secondary Subject Heading: | Nutrition and metabolism, Public health, Health policy |
| Keywords: | PAEDIATRICS, NUTRITION & DIETETICS, Paediatric endocrinology < PAEDIATRICS |
| | |

SCHOLARONE™
Manuscripts

# Are food and drink retailers within NHS venues adhering to NICE Quality Standard 94 guidance on childhood obesity?

List of authors:

A James[1], L Birch[2], P Fletcher[3], S Pearson[4], C Boyce[5], AR Ness[2], J Hamilton-Shield[2], FE Lithander[2]

Corresponding author: Fiona Lithander, fiona.lithander@bristol.ac.uk

Keywords: childhood, obesity, NICE Quality Standard 94, retailers, food, drink

For peer review only

## Abstract

**Objective**

To assess whether the food and drink retail outlets in two major National Health Service (NHS) district general hospitals in England adhere to Quality Statements 1-3 of the UK National Institute for Health and Care Excellence (NICE) Quality Standard 94.

**Design**

Cross-sectional, descriptive study to assess the food and drink options available in vending machines, restaurants, cafes and shops in two hospitals.

**Main outcome measures**

Adherence to Quality Statement 1 whereby the food and drink items available in the vending machines were classified as either healthy or less healthy using the Nutrient Profiling Model (NPM). Compliance with Quality Statements 2 and 3 was assessed through the measurement of how clearly the shops, cafes and restaurants displayed nutrition information on menus, and the availability and prominent display of healthy food and drink options in retail outlets, respectively.

**Results**

Adherence to Quality Statement 1 was poor. Of the 18 vending machines assessed, only 7 (39%) served both a healthy food and a healthy drink option. Neither hospital was compliant with Quality Statement 2 wherein nutritional information was not available on menus of food providers in either hospital. There was inconsistent compliance with Quality Standard 3 whereby healthy food and drink options were prominently displayed in the two main hospital restaurants, but all shops and cafes prioritised the display of unhealthy items.

**Conclusions**

Neither hospital was consistently compliant with Quality Statements 1-3 of the NICE Quality Standard 94. Improving the availability of healthy foods and drinks whilst reducing the display and accessibility to less healthy options in NHS venues may improve family awareness of healthy alternatives. Making it easier for parents to direct their children to healthier choices is an ostensibly central component of our health care system.


**Strengths and limitations of this study**

- This is the first study to evaluate the consumer nutrition environment of two NHS hospitals in England, and to assess their compliance to recommendations of the NICE Quality Standards 94.
- Only two hospitals in the same geographical area in England were assessed.
- The inclusion of a second assessor to provide an additional independent evaluation would have been beneficial to reduce possible bias.



For peer review only - http://bmjopen.bmj.com/site/about/guidelines.xhtml

**Introduction**

Childhood obesity is a global health concern[1]. 9.3% of 4-5 year olds and 19.8% of 10-11 year olds in the UK are currently classified as obese [2, 3] which is known to increase morbidity and mortality in later life through its association with cancer, cardiovascular disease and mental health disorders.[3, 4] The doubling of obesity rates between the first and final years of primary school highlights early childhood as a high-risk period for obesogenic factors to take effect[2, 3]. Whilst overweight and obese children are more likely to become obese adults,[4] healthy weight adults who were obese during childhood have similar risk for metabolic diseases as those who maintained a healthy weight throughout life[5], suggesting that childhood is an opportune time for obesity prevention.

In addition to treating ill health, the National Health Service (NHS) Constitution pledges to maintain wellbeing through 'supporting individuals to promote and manage their own health[6]. The NICE Quality Standard 94 entitled 'Obesity in children and young people: prevention and lifestyle weight management programmes'[4] (Table 1) identifies NHS venues as important settings in which to implement childhood obesity prevention strategies. Children may attend an NHS venue such as a hospital as a patient or a visitor and can be directly or indirectly influenced by the consumer nutrition environment within a hospital.

There is increasing evidence that the consumer nutrition environment is an important determinant of dietary behaviour and obesity[7-9]. The consumer nutrition environment describes what an individual encounters when he or she enters a venue where food is purchased or consumed[10]. Studies have shown that healthier eating patterns have been developed by students when their school nutrition environments were improved through an increase in the nutrient quality of the food provided[11, 12]. Moreover, evidence suggests that hospital retailers such as shops, cafes and restaurants can serve as examples of healthy nutrition environments[13]. Studies conducted in Canada and the USA have assessed consumer nutrition environments in hospitals and have shown that food and drink retailers in both adult [14, 15] and children's [13, 16] hospitals offer unhealthy food and drink options. Little is known however about the consumer nutrition environment of NHS venues in England and in addition, few tools exist to allow it to be assessed.

The aim of the current study was to measure compliance to National Institute for Health and Care Excellence (NICE) Quality Standard 94 by assessing the consumer nutrition environment of two NHS District General Hospitals in England. An assessment tool was developed to guide quality assessment of food and drink provision in these two NHS venues.



For peer review only - http://bmjopen.bmj.com/site/about/guidelines.xhtml

## Methods

*Setting/Hospitals*

This study was conducted in two major District General Hospitals in the South West of England in July 2016. These two NHS hospitals were chosen as the lead author was studying in these hospitals during this period. Both hospitals offer paediatric, day-time access for assessment and out-patients but only one, the larger hospital, provides inpatient care. One Hospital has approximately 680 beds in total, 8 vending machines and a total of 6 food retail outlets which comprise 1 restaurant, 3 cafes and 2 shops. The other has approximately 380 beds, 10 vending machines and 7 food retail outlets comprising 1 restaurant, 2 cafes and 3 shops. The two restaurants served hot and cold meals throughout the day. The only meals served by the cafes were sandwiches and salads in addition to confectionary, savoury snack items, and hot drinks. Food and drink retailers in each hospital were evaluated against Quality Statements 1-3 of NICE Quality Standard 94 (Table 1). Data were collected by the lead author during a 2 week period in July 2016 where each vending machine and retail outlet was visited by her on one occasion only.

*Assessment Tool*

The Consumer Nutrition Environment Tool (C-NET) was developed by the lead author. To measure adherence to Quality Statement 1, the user recorded each food and beverage item for sale in each of the vending machines. The nutrient composition for each item was then retrieved from the item packaging or from product nutrition information available online. The UK Department of Health Nutrient Profiling Model (NPM) was used to classify these foods and drinks into two categories; less healthy and healthy, and details of this model and the scoring system have been published elsewhere[17]. To do this, the nutrient content of each food and beverage was assessed against a set of published criteria to determine whether it contains certain nutrients above or below particular thresholds. The NPM identifies foods and drinks that are high in fat, salt or sugar and enables them to be differentiated into two categories, healthy or less healthy, based on their nutrient composition. The NPM uses a scoring system where points are allocated on the basis of the nutrient content of 100g of a food or beverage. Points are awarded for energy, saturated fat, total sugar and sodium ('A' nutrients) and fruit, vegetable and nut content, fibre and protein ('C' nutrients). The score for 'C' nutrients is subtracted from the 'A' nutrients score to give a final NPM score. Foods that score four points or more, and drinks which score one or more points are classified as 'less healthy' using the NPM. An example is that of raw nuts which had a nutrient composition (per 100g) as follows: 2656kJ, 9.1g saturated fat, 3.7g total sugar, 0.02g salt; this item scored 16 'A' points. Upon calculation of 'C' points for the raw nuts (per 100g), they contained >80% fruit, vegetables or nuts, 6g fibre, and 15.8 g protein, giving it a total of 5 'C' points. The final score for this item was 1, hence this item was classified as healthy according to the NPM. The price of each food and beverage, and the number of vending machines within each NHS venue where each food and



For peer review only - http://bmjopen.bmj.com/site/about/guidelines.xhtml

beverage was available was also recorded. Data collected were used to measure compliance with Quality Statement 1.

The NPM was not used to measure compliance with Quality Statements 2 or 3; this was done using a more subjective assessment of the consumer nutrition environment by the lead author. To measure adherence to Quality Statements 2 and 3, she evaluated the quality of the consumer nutrition environment within each hospital restaurant, café or shop through answering a series of yes/no questions which centred around 3 main themes: the provision of nutrient information for meals on menus, advertising and promotions and the prominent placing of healthy and unhealthy items in the retail outlets (Table 3). For Quality Statement 2, the lead author examined the retail outlets for the display of nutritional information on menus. For Quality Statement 3, her knowledge of the Eatwell Guide[18] was used whereby foods within the food groups such as fruit, vegetables, dairy, wholegrain and high fibre foods, eggs, lean meat and pulses, were classified as healthy, and foods and drinks which are high in fat, salt and sugars such as biscuits, sweets, chocolate, and sugar sweetened beverages were classified as unhealthy.

*Procedure*

Compliance with Quality Statement 1 was observed if a NHS venue had a vending machine where at least one healthy food and at least one healthy beverage option were available for sale in the same machine. Compliance with Quality Statement 2 was observed if retail outlets provided information on energy, total fat, saturated fat, salt and sugar content of meals and snacks. Quality Statement 2 states that listing ingredients and cooking methods constitutes an acceptable level of nutritional information if the information on energy, fat, saturated fat, salt and sugar content is not available. Drinks were not assessed as part of Quality Statement 2 since the rationale provided by NICE refers to food only. When nutrient information was not clearly displayed, the lead author liaised with members of catering staff to identify whether this information would be available on request. With respect to Quality Statement 3, the lead author examined whether healthy and less healthy foods and drinks were advertised outside the venues, whether advertising stalls containing food and drink items were placed nearest the door of the shop, café or restaurant, whether items were at a child's eye level which was classified as 1 metre height, and whether items were displayed at the payment area. Compliance with Quality Statement 3 was observed if healthy food and drink choices were displayed prominently in the retail outlets and poor compliance was deemed if unhealthy options were prominently displayed. The definition of compliance with the three Quality Statements was selected by the authors given the lack of guidance regarding what constituted compliance provided by NICE Quality Standard 94.

*Data analyses*

For peer review only



For peer review only - http://bmjopen.bmj.com/site/about/guidelines.xhtml

Data on the NPM scored are presented as mean (sd) and range. Qualitative data were initially coded and collated into themes by the lead author. Interpretation of these data and the identification of themes was reviewed and discussed by multiple authors (AJ, LB, FEL) throughout the process to validate the findings.

For peer review only



For peer review only - http://bmjopen.bmj.com/site/about/guidelines.xhtml

**Results**

Two restaurants, 5 cafes, 5 shops and 18 vending machines were included in the analyses across both hospital sites. Data were originally collected from 29 vending machines but 11 were excluded from analyses. Ten vending machines, which sold hot drinks only, were excluded because nutrition information was not clearly displayed nor was it available online or from the supplier when requested by the lead author. One vending machine, which sold only frozen items such as burgers, was also excluded because this machine was removed from the hospital before the end of the data collection period.

*Quality Statement 1: "Children and young people, and their parents or carers, using vending machines in local authority and NHS venues can buy healthy food and drink options"*

Of the 18 vending machines, 7 (39%) offered both a healthy food and a healthy drink (Table 2). 55 different items were on sale across all 18 vending machines, 40 of which were foods and 15 were drinks. Where the same drink was served in a 330ml can or in a larger 500ml bottle, this was classified as two separate drinks. When the 40 foods alone were assessed using the NPM, the mean NP score was 18.3, (9.3). Only 4 of the 40 (10%) foods were classified as healthy with a NP score of less than 4; these items were baked crisps, a packet of dried fruit and nuts, a packet of raw nuts and a muesli bar (NP score -1.3 (sd 4.5)). When the other 36 (90%) food items were analysed according to the NPM, the NP score was 20.5 (sd 6.8), significantly greater than the cut-off for the less healthy classification of 4 points or more. The 4 healthy foods items were priced equivalently to similar less healthy items available in the vending machines.

When the drinks were assessed, where healthy is defined as scoring 1 point or more, 8 of the 15 (53%) available were classified as healthy (NP score -0.5 (sd 1.4)) using the NPM cut-off of 1 point or more, and the remainder classified as less healthy (NP score 1.7 sd (0.5)). The drinks that were classified as healthy were an orange flavoured sugar sweetened beverage, concentrated orange juice, bottled water, and sugar-free cola drinks. The healthy options were marginally less expensive (£1.25 sd 0.27p) than the less healthy options (£1.36 sd 0.24p).

*Quality Statement 2: "Children and young people, and their parents or carers, see details of nutritional information on menus at local authority and NHS venues"*

Neither hospital was compliant with Quality Statement 2. When the two restaurants were analysed against Quality Statement 2, it was found that neither displayed nutrition information on their menus for hot or cold meals. When the lead author asked the retail staff, she was informed that this information was available only for cold fillings offered with jacket potatoes and for a proportion of the filled sandwiches on sale. Quality Statement 2 states that if the nutrient content of a recipe is unavailable to consumers, the ingredients and cooking methods should be available. Whilst this information was not displayed on menus, catering staff

For peer review only

advised that this information would be available on request, though it was not specifically requested by the lead author as part of this study. Though not displayed on menus, a range of sandwiches and salads that were made onsite were available in the restaurants. Nutritional information detailing total energy, fat, saturated fat, sugar and salt content was displayed on the labels of these items.

None of the cafes provided nutrient information on their menus, meaning that all five cafes were not compliant with Quality Statement 2. However, three of the cafes offered pre-packaged sandwiches and comprehensive nutrition information was available on the packaging of these. The remaining two cafes sold sandwiches, baguettes and salads made by a local catering company. Catering staff in these cafes advised that specific nutrition information was not available for these items but that details of ingredients were available on request, though the lead author did not specifically request this information. Quality Statement 2 was not relevant to the shops as they did not have menus.

*Quality Statement 3: "Children and young people, and their parents or carers, see healthy food and drink choices displayed prominently in local authority and NHS venues".*

There was inconsistent compliance to Quality Statement 3 in both hospitals. When the two restaurants were analysed, adherence to Quality Statement 3 varied. One of the restaurants housed a stall inside the entrance, which sold fresh fruit, vegetables, and local produce and was labelled as a 'Farm Shop'. The same restaurant also sold steamed potatoes and these, in addition to fresh vegetables, were clearly advertised as a cheaper option than a portion of chips. In the second restaurant there were several signs which advertised 'Healthier Options'. This included a 'lighter breakfast' which comprised grapefruit segments, natural yoghurt, a bowl of breakfast cereal with milk, a pastry and a cheese portion. Healthier snack options such as fresh fruit were prominently displayed on service counters and by the payment area. All five cafes demonstrated good adherence to Quality Statement 3. The balance of healthy and less healthy items on display in the most prominent areas such as the queuing or payment area was equal and there was less evidence of advertising and price promotions on less healthy items than in the shops. Fresh fruit was available beside the payment areas in 4 of the 5 cafes. However baked goods such as muffins, scones, cookies and cakes were displayed in glass cabinets or on the counter surface next to the queuing and payment area in all 5 cafes.

Healthy options made up approximately 25% of the food and beverage items available in the shops and these items were displayed in less prominent locations such as at the back of the shop or on low-lying shelves. Posters were used in all of the hospital shops and cafes to advertise price promotions on less healthy options including signs placed outside shops and cafes to advertise items such as hot dogs and ice cream. Cakes made onsite were displayed next to the payment area in 2 of the shops, and less healthy options including



For peer review only - http://bmjopen.bmj.com/site/about/guidelines.xhtml

227 muffins and chocolate bars were displayed at the payment areas in all shops and cafes. Price promotions were advertised for several of these less healthy products displayed at the payment area.

230 The layout of the unhealthy items in many of the shops appeared to target children. The 'pic n mix' sweet stalls in both of the largest shops had prominent positions near the entrance and products such as colourful sugar-sweetened iced drinks, lollipops and chocolate eggs containing small toys were displayed at the payment area or at a child's eye level. There was a display in one shop with a sign which read 'Big Kids Sweet Zone' offering 35 different sweets all priced at less than £1. Both of the largest shops used posters to advertise a price deal on fresh fruit, however fruit stalls were not in a prominent location and were poorly stocked.

For peer review only



For peer review only - http://bmjopen.bmj.com/site/about/guidelines.xhtml

**Discussion**

This study is the first to describe the consumer nutrition environment of two NHS hospitals in England and to assess their compliance with NICE Quality Standard 94. We found that food and drink retailers in these two hospitals demonstrated poor compliance with this Quality Standard. Only 39% of vending machines across both hospitals served both one healthy food and one healthy drink option indicating poor compliance with Quality Statement 1. Moreover, 90% (36/40) of the food items in the vending machines were classified as less healthy suggesting that the consumer may have difficulty identifying and locating the 10% of items classified as healthy. 53% (8/15) of the drink items available were classified as unhealthy. It was found that the two hospitals were not compliant with Quality Statement 2, which refers to the availability of nutritional information at the point of choosing food or drink. Compliance was variable in relation to Quality Statement 3, where restaurants engaged in various activities in the display of healthy options yet all cafes and shops favoured the prominent display and advertising of unhealthy foods and drinks.

Hospitals have a role to play in advocating for healthy lifestyle and good nutrition[19, 20]. The consumer nutrition environment in 14 children's hospitals in California was assessed and it has been suggested that nutrition intervention is needed to improve the availability of healthy food and beverage options[13]. The authors of that research suggest that inexpensive interventions could be used such as providing nutrient information and introducing signage that promotes healthy choices[13]. McDonald and co-workers[16] reported that university-affiliated children's hospitals in Canada and the US provide a suboptimal health environment, and hypothesise that a reliance on revenue from outlets which provide less nutritious foods may be a factor. Such results are not restricted to children's hospitals however. Winston and co-workers[14, 15]described the nutrition environment of 39 hospitals in the USA, and found that the consumer nutrition environment was poor and suggested that dietary interventions are justified in health settings.

The current study found that only 39% of vending machines provided both a healthy food and a healthy drink, and yet this result must be interpreted with caution. The most widely available healthy food item was baked crisps which were found in 33% vending machines, and when presented in the same vending machine as a healthy drink such as bottled water or a sugar-free drink, the machine, and by extension the hospital, was classified as compliant with Quality Statement 1. It is known that nutrient profile schemes have become drivers for product reformulation[17] and the baked crisps, and indeed the orange flavoured sugar sweetened beverage which was classified as healthy, may have been reformulated to meet the NPM criteria. It is noteworthy that both of these items are classified as unhealthy according to the Eatwell Guide[18] where they are both in the category of foods which are high in fat, salt and sugar yet are classified as healthy when the NPM was used.



For peer review only - http://bmjopen.bmj.com/site/about/guidelines.xhtml

It is well accepted that nutrition information at the point of purchase can influence food choice[21]. Consumers under-estimate by 2 to 4 times the saturated fat, energy and sodium content of restaurant foods[21], yet providing accurate point-of-sale nutrition information is known to improve consumer choice[21]. None of the restaurants, cafes or shops examined in the current study were compliant with Quality Statement 2, which states that nutrition information should be available for consumers at the point of choosing food and drink options. It is a hospital's duty to empower consumers with the information required to make an informed choice[22]. When asked, staff suggested that nutrition information would be provided to consumers on request yet the format of this information, exactly when it would be provided, and how user-friendly it would be, is unknown.

The availability and accessibility of unhealthy foods have been identified as risk factors for overeating[23] and it is understood that the prominence of food and drinks displays can influence consumer choice[24, 25]. In the current study, the restaurants demonstrated variable compliance to Quality Statement 3 which states that healthy food and drink options are displayed prominently. Evidence of good practice included one restaurant which advertised fresh fruit and vegetables at competitive prices, whilst the other advertised what was referred to as a 'lighter breakfast'. This option, however, also included a pastry and cheese portion, both of which are known to be high in fat, saturated fat and salt. The 5 shops and 5 cafes all prioritised the prominent display of unhealthy options, and whilst fruit was available beside the payment area in 4 of the 5 cafes albeit poorly stocked, so too were unhealthy baked goods which were displayed by the payment area in all 5 cafes. Research has shown that unhealthy food and drink items are difficult for consumers to avoid in supermarkets, as not only do they take up more shelf space than healthy items such as fruit and vegetables, but are more often displayed at payment areas, as seen in the current study[26-28]. Moreover, the placement of healthy food items at a payment area can lead to a substantial positive impact on sales of these products[29]. In addition, both of the largest shops in the current study displayed an array of sweets, chocolate and sugar-sweetened drinks at child's eye level, and evidence suggests that placing products on shelves at eye-level positively influences sales[25].

The Consumer Nutrition Environment Tool (C-NET) used in the current study was developed by the lead author of this study. Whilst this tool has not been validated, it allowed both the objective collection of data using the NPM, and the subjective assessment of marketing practices that contribute to food purchases using a series of questions (Table 3). Other studies which have assessed food retailers in hospitals have used a variety of methods; some relied on telephone interviews with cafeteria directors which may have biased the data collected[16] whilst others used a validated tool such as the University of Pennsylvania Nutrition



For peer review only - http://bmjopen.bmj.com/site/about/guidelines.xhtml

Environment Measures Scale (NEMS). However, no equivalent British tool was found and thus the lead author developed the C-NET. When compliance with Quality Statement 1 was assessed in the current study, C-NET used the NPM which was developed by the Food Standards Agency in 2004-2005, and was subject to rigorous scientific scrutiny, extensive consultation, and review. It is supported by the independent Scientific Advisory Committee on Nutrition and a wide range of nutrition experts[30]. The NPM was introduced as mandatory in 2007 by the UK Office of Communications and DH to regulate food and drinks in the context of television advertising to children. This model was selected for use in the current study as it is widely regarded as scientifically robust and effective in identifying less healthy items, and in practical terms, it is well established in the UK. There are other government endorsed NPMs available, such as the EU Pledge model and the WHO Europe model, but these use multiple categories and subcategories of foods which have raised concerns of ambiguity and additional complexity that may reduce the clarity and transparency of the models[31]. They also do not have the same track record of effective use in the UK regulatory environment. The Committee of Advertising Practice (CAP), the lead UK organisation that write and maintain the UK Advertising Codes to ensure advertising in the UK is legal, decent, honest and truthful, have adopted the DH NPM in their recently published regulatory statement on food and soft drink advertising to children, which was developed following public consultation and will come into effect in the UK on 1 July 2017[32].

Consumer organisations have set up campaigns in the UK and Australia asking supermarkets to remove unhealthy food and drink items from payment and queuing areas[33-35] but it is not known if such campaigns exist for retails outlets in hospitals. NICE Quality Standards are a set of prioritised statements, which draw on existing guidance to set out the priority areas for quality improvement in health and social care. They are designed to drive measurable quality improvements yet, using them in the current study has proven challenging. Quality Statement 1 refers to the availability of 'healthy food and drink options' in vending machines, yet nowhere in NICE Quality Standard 94 or in the associated documents is there a clear definition of what 'healthy' means, and it was on this basis that the DH NPM was chosen as a method of classification. Moreover, the definition of compliance to Quality Statement 1, where a vending machine contained both a healthy food and a healthy drink was chosen by the study authors given the lack of guidance on what constitutes as compliance from NICE Quality Standard 94. The authors considered compliance to Quality Statements 2 and 3 if most retail outlets in the hospitals adhered to the respective statement. 'Evidence' of 'arrangements to display healthy food and drink options in prominent places' constitutes adherence to Quality Statement 3 according to NICE Quality Standard 94. However, challenges arose in assessing what constituted a 'healthy option' and a 'prominent place', and was subjective. The measurement of adherence to all three Quality Statements is open to interpretation, and assessment of adherence was a challenging task given their non-quantifiable and non-specific nature.


For peer review only - http://bmjopen.bmj.com/site/about/guidelines.xhtml

The main limitation of the current study is that only two hospitals were included, both of which are in the same geographical area in England. Moreover, a second assessor would have provided an additional independent evaluation of the offerings. Further research is needed on a more representative sample of hospitals to fully understand compliance to NICE Quality Standard 94. However, such a study would be subject to similar limitations in terms of the vague nature of this Quality Standard and a more objective means of assessing adherence to the Statements within the Quality Standard would be necessary.

In conclusion, the current study showed that two NHS hospitals demonstrated poor compliance with Quality Statements 1-3 in NICE Quality Standard 94. The lack of availability of healthy foods and drinks, the absence of nutritional information on the menus, the lack of advertising and display of healthy items and the consistent advertising and prominent display of unhealthy items highlights that improvements are required in NHS venues such as hospitals. These findings have been shared with the NHS Trust of one of these hospitals and re-evaluation will take place in the future. However, as the prevalence of childhood obesity continues to rise globally, it is important that every opportunity is taken to improve the nutrition environment for children's food choices. Hospitals have a duty to provide consumers with the information required to make informed nutrition choices and should take the lead in supplying food and drinks that reflect evidence based nutrition[22].

For peer review only



For peer review only - http://bmjopen.bmj.com/site/about/guidelines.xhtml

Author affiliations:

[1]5th year medical student, Faculty of Health Sciences, University of Bristol

[2]National Institute for Health Research Biomedical Research Centre, Bristol (Nutrition Theme), University Hospitals Bristol NHS Foundation Trust and the University of Bristol, UK

[3]Department of General and Old Age Medicine, Cheltenham General Hospital, Gloucestershire, UK,

[4]Department of Clinical Strategy, Gloucestershire Hospitals NHS Foundation Trust Main Board, Gloucestershire, UK

[5]Department of Clinical Strategy, Gloucestershire Hospitals NHS Foundation Trust, Gloucestershire, UK

Acknowledgements

The authors thank Amanda Chong for assistance in the preparation of the manuscript and acknowledge support from the National Institute of Health Research (NIHR) Bristol Nutrition Biomedical Research Unit.

Contributors

AJ and JHS designed and collected the data. PF, SP and CB supervised and oversaw AJ during the data collection period. LB calculated and analysed the nutrient model scores. FEL, LB and ARN supervised the data analyses and prepared the manuscript. All authors revised the manuscript and approved the final version.

Funding

This research received support from the from the National Institute of Health Research (NIHR) Bristol Nutrition Biomedical Research Unit.

Data sharing

No additional data are available.

Competing interests

No competing interests.

For peer review only



For peer review only - http://bmjopen.bmj.com/site/about/guidelines.xhtml

**References**


1. Lobstein T, Jackson-Leach R, Moodie ML, *et al.* Child and adolescent obesity: part of a bigger picture. *Lancet*. 2015 Jun 20;385(9986):2510-20.
2. NHS Digital. *National Child Measurement Programme England, 2015/16 school year*. 2016. https://www.gov.uk/government/statistics/national-child-measurement-programme-england-2015-to-2016-school-year (accessed 01 Dec 2016).
3. Public Health England. *About Obesity The National Obesity Observatory*. 2016. http://www.noo.org.uk/NOO_about_obesity (accessed 31 November 2016).
4. National Institute for Health and Care Excellence. *Obesity in children and young people: prevention and lifestyle weight management programmes. Quality Standard 94 (QS94).* 2015. https://www.nice.org.uk/guidance/qs94 (accessed 31 Oct 2016).
5. Juonala M, Magnussen CG, Berenson GS, *et al.* Childhood adiposity, adult adiposity, and cardiovascular risk factors. *N Engl J Med*. 2011;365(20):1876-85.
6. Department of Health. *NHS Constitution for England*. 2012. https://www.gov.uk/government/publications/the-nhs-constitution-for-england (accessed 31 Oct 2016).
7. Black C, Ntani G, Inskip H, *et al.* Measuring the healthfulness of food retail stores: variations by store type and neighbourhood deprivation. *Int J Behav Nutr Phys Act*. 2014;11:69.
8. Caspi CE, Sorensen G, Subramanian SV, *et al*. The local food environment and diet: a systematic review. *Health Place*. 2012;18(5):1172-87.
9. Holsten JE. Obesity and the community food environment: a systematic review. *Public Health Nutr*. 2009;12(3):397-405.
10. Glanz K, Sallis JF, Saelens BE, *et al*. Healthy nutrition environments: concepts and measures. *Am J Health Promot*. 2005;19(5):330-3.
11. Katz DL, O'Connell M, Yeh MC, *et al*. Public health strategies for preventing and controlling overweight and obesity in school and worksite settings: a report on recommendations of the Task Force on Community Preventive Services. *MMWR Recomm Rep*. 2005;54(RR-10):1-12.
12. Economos CD, Hyatt RR, Goldberg JP, *et al.* A community intervention reduces BMI z-score in children: Shape Up Somerville first year results. *Obesity (Silver Spring).* 2007;15(5):1325-36.
13. Lesser LI, Hunnes DE, Reyes P, *et al.* Assessment of food offerings and marketing strategies in the food-service venues at California Children's Hospitals. *Acad Pediatr*. 2012;12(1):62-7.
14. Winston CP, Sallis JF, Swartz MD, *et al*. Reliability of the hospital nutrition environment scan for cafeterias, vending machines, and gift shops. *J Acad Nutr Diet*. 2013 ;113(8):1069-75.
15. Winston CP, Sallis JF, Swartz MD, *et al*. Consumer nutrition environments of hospitals: an exploratory analysis using the hospital nutrition environment scan for cafeterias, vending machines, and gift shops, 2012. *Prev Chronic Dis*. 2013;10:E110.
16. McDonald CM, Karamlou T, Wengle JG,*et al*. Nutrition and exercise environment available to outpatients, visitors, and staff in Children's hospitals in Canada and the United States. *Arch Pediatr Adolesc Med*. 2006;160(9):900-5.
17. Rayner M, Scarborough P, Lobstein T. *The UK Ofcom Nutrient Profiling Model: Defining 'healthy' and 'unhealthy' foods and drinks for TV advertising to children*. 2009. https://www.ndph.ox.ac.uk/cpnp/about/publications-and-reports/group-reports/uk-ofcom-nutrient-profile-model.pdf (accessed 31 Oct 2016).
18. Public Health England. *The Eatwell Guide 2016*. 2017. https://www.gov.uk/government/publications/the-eatwell-guide (accessed 31 Oct 2016).
19. Cram P, Nallamothu BK, Fendrick AM, *et al*. Fast food franchises in hospitals. *JAMA*. 2002;287(22):2945-6.
20. Trinidad C. McDonald's joins Childrens Hospital of Los Angeles. *Calif Hosp*. 1990;4(1):39-40.
21. Burton S, Creyer EH, Kees J, *et al*. Attacking the obesity epidemic: the potential health benefits of providing nutrition information in restaurants. *Am J Public Health*. 2006;96(9):1669-75.
22. Freedhoff Y, Stevenson R. Frying up hospital cafeteria food. *CMAJ*. 2008;179(3)213-4.
23. Brownell K. Does a "toxic" environment make obesity inevitable? *Obes Manag*. 2005;1(2):52-5.




For peer review only - http://bmjopen.bmj.com/site/about/guidelines.xhtml

24. Inman JJ, Winer RS, Ferraro R. The interplay among category characteristics, customer characteristics, and customer activities on in-store decision making. *J Mark.* 2009;73(5):19-29.
25. Chandon P, Hutchinson JW, Bradlow E, *et al*. Does in-store marketing work? Effects of the number and position of shelf facings on brand attention and evaluation at the point of purchase. *J Marketing*. 2009;73(6):1-17.
26. Farley T, Rice J, Bodor JN, *et al*. Measuring the food environment: shelf space of fruits, vegetables, and snack foods in stores. *J Urban Health*. 2009;86(5):672-82.
27. Thornton LE, Cameron AJ, McNaughton SA, *et al.* Does the availability of snack foods in supermarkets vary internationally? *Int J Behav Nutr Phys Act*. 2013;10(56).
28. Gebauer H, Laska MN. Convenience stores surrounding urban schools: an assessment of healthy food availability, advertising, and product placement. *J Urban Health*. 2011;88(4):616-22.
29. Sigurdsson V, Larsen NM, Gunnarsson D. Healthy food products at the point of purchase: an in-store experimental analysis. *J Appl Behav Anal*. 2014;47(1):151-4.
30. Department of Health. *The nutrient profiling model*. 2011. https://www.gov.uk/government/publications/the-nutrient-profiling-model (accessed 25 Oct 2016).
31. Brinsden H, Lobstein T. Comparison of nutrient profiling schemes for restricting the marketing of food and drink to children. *Pediatr Obes*. 2013;8(4):325-37.
32. Committee of Advertising Practice (CAP). *CAP Consultation: food and soft drink advertising to children*. 2016. https://www.asa.org.uk/asset/98337008-FA03-481B-92392CB3487720A8/ (accessed 30 Nov 2016).
33. Piacenti M, MacEadyen L, Eadie D. Corporate social responsibility in food retailing. *Int J Retail Distrib Manag*. 2000;28(11):459-69.
34. Sustain. *Press for change at the till: Get the junk off the checkouts!* 2014. https://www.sustainweb.org/childrensfoodcampaign/chuck_junk/ (accessed 31 Oct 2016).
35. Parent's Voice. *Healthy checkouts*. 2014. http://parentsvoice.org.au/our-work/healthy-checkouts/ (accessed 25 Oct 2016).

For peer review only

Table 1: NICE Quality Standard 94 "Obesity in children and young people: prevention and lifestyle weight management programmes"

| | Quality Statement | How it was assessed and how compliance was defined |
|---|---|---|
| Quality Statement 1 | Children and young people, and their parents or carers, using vending machines in local authority and NHS venues can buy healthy food and drink options | Foods and drinks were classified as healthy or less healthy using the NPM. A hospital was deemed compliant if it housed one vending machine which contained both one healthy food and one healthy drink |
| Quality Statement 2 | Children and young people, and their parents or carers, see details of nutritional information on menus at local authority and NHS venues. | A hospital was deemed compliant if the majority of its retails outlets displayed nutritional information on their menus |
| Quality Statement 3 | Children and young people, and their parents or carers, see healthy food and drink choices displayed prominently in local authority and NHS venues | Foods and drinks were defined as healthy if they were within the four food groups in the Eatwell Guide. A hospital was deemed compliant if the majority of its retail outlets prominently displayed healthy food and drinks options |

NPM, nutrient profile model[17]

For peer review only

Table 2: Mean (sd) nutrient profile model scores for all 40 foods and 15 drinks found in the vending machines; data includes healthy and less healthy foods and drinks. A food is classified as less healthy where it scores ≥4 points. A drink is classified as less healthy where it scores ≥ 1 point

| | Mean | sd | Range |
|---|---|---|---|
| Crisps, n=10 | 10.3 | 4.3 | 1, 17 |
| Chocolate, n=19 | 25.6 | 1.3 | 23, 27 |
| Sweets, n=2 | 15.5 | 0.7 | 15, 16 |
| Sweet & savoury biscuits, n=4 | 19.8 | 6.7 | 10, 25 |
| Dried fruit and /or nuts, n=5 | 6.4 | 12.8 | -8, 25 |
| Drinks, n=15 | 0.5 | 1.6 | -4, 2 |

For peer review only

Table 3: Consumer Nutrition Environment Tool (C-NET); the questions were asked to ascertain if retail outlets adhered to Quality Statements 2 and 3. Questions around nutritional information for hot and cold meals relate to Quality Statement 2. Questions around advertising and promotions, and the layout & prominent placing of healthy and unhealthy items relate to Quality Statement 3.

| |
|---|
| ***Nutritional information for hot and cold meals*** |
| Are comprehensive details on nutritional content available for sandwiches, salads and other cold and packaged meal options? |
| If this information is not provided, are the ingredients listed? |
| Are comprehensive details on nutritional content available for hot meal options? |
| If this information is not provided, are details provided about the ingredients and cooking methods? |
| Are posters or labels used to direct consumers to healthier meal options? |
| ***Advertising & Promotions*** |
| Are signs or other promotional materials used to advertise healthy options? |
| Are signs or other promotional materials used to promote unhealthy options? |
| Are there advertisements for unhealthy items that are clearly aimed at children? For example the use of bright colours, television/cartoon characters and/or specific wording of advertisement? |
| Are price incentives (e.g. reduced price offers) for healthy items clearly displayed (e.g. using signs)? |
| Are price incentives (e.g. reduced price offers) for unhealthy items clearly displayed (e.g. displayed at payment area)? |
| ***Layout & prominent placing of healthy & unhealthy items*** |
| Are healthy food and drink items promoted via prominent placing e.g. near the entrance to a shop or as part of a large/attractive display? |
| Are unhealthy food & drink options promoted via prominent placing? |
| Are unhealthy options displayed at the payment area or point of purchase? |
| Are unhealthy options displayed at an easy height for children to 'grab' (1 metre height) |

For peer review only

Table 4. Observed activities which shops, cafes and restaurants engaged in against which compliance to Quality Statement 3 was measured. The number in parentheses is number of shops or cafes or restaurants that engaged in this activity

| | Quality Statement 3: Prominent placing of healthy options | |
|---|---|---|
| | **Compliant** | **Non-compliant** |
| Shops, n=5 | ***Advertising & Promotions***<br>• 'Meal-deal' promotion includes sugar-free drink options (5)<br>• Poster advertising price promotions on fresh fruit and dried fruit (2) | ***Advertising & Promotions***<br>• Fruit available was not reflective of the fruit advertised on the poster (2)<br>• 'Big Kids Sweet Zone' offering 35 different products at 'pocket money' prices (1)<br>• Posters advertising price promotions on unhealthy options including share-size confectionary, sugar-sweetened beverages, crisps, 'coffee & muffin deal' hotdogs and ice-cream (5)<br>• 'Low saturated fat' sandwich range available but not advertised (2)<br>• 'Meal-deal' poster advertising sandwich, crisps or piece of fruit plus a drink although the fruit option was unavailable in some shops (3)<br><br>***Layout & prominent placing of healthy & unhealthy items***<br>• Fresh fruit stall located at the back of the shop (1)<br>• Bruised fruit (1)<br>• First visible items on entry were pic'n'mix stalls, confectionary and crisps (5)<br>• Floor-to-ceiling confectionary stall adjacent to the entrance (1)<br>• Multiple rows of confectionary at 1m height (5)<br>• Ice-cream freezer at the entrance and visible from main hospital concourse (2)<br>• Homemade cakes prominently displayed at payment areas (2)<br>• Price promotions on unhealthy snack items prominently displayed at payment areas (2) |

For peer review only

| | | |
|---|---|---|
| Cafes, n=5 | ***Advertising & Promotions***<br>• Poster advertising healthier wraps (roast chicken rainbow/sweet potato/falafel) (1)<br>• Skimmed/semi-skimmed/soya milk advertised as options for drinks & porridge (1)<br>• Poster and table-top advertisements for fruit smoothies containing 100% fruit in 500ml portions stating '1 of your 5 a-day' (1)<br><br>***Layout & prominent placing of healthy & unhealthy items***<br>• 'Healthier range' wraps/sandwiches/salads available and clearly labelled (1)<br>• Healthy breakfast options clearly displayed. Options included porridge pots, fruit salad and yoghurt (2)<br>• Fresh fruit salad tubs prominently displayed in glass display cabinet (1)<br>• Salads served without dressing. Self-service dressings available (1)<br>• Prices available for soup with or without a bread roll (1)<br>• 2 healthier snack options prominently displayed which were cereal bars and healthier biscuits (2)<br>• Fresh fruit prominently displayed although no price information was available (1)<br>• Baked crisps available (2) | ***Advertising & Promotions***<br>• Price promotions on baked goods such as brownies displayed prominently next to the payment area (1)<br>• Poster advertisement in main hospital foyer outside café for 'double chocolate cookie mocha creamy cooler' (1)<br>• Poster advertisement for 'Product of the month' which were cookies (1)<br><br>***Layout & prominent placing of healthy & unhealthy items***<br>• Cakes and other baked goods prominently displayed at service and payment areas (5)<br>• No healthy snacks or fruit available (1)<br>• Crisps and salted nuts prominently displayed next to café entrance and in front of healthier snack items (2)<br>• Large chocolate bar selection available (5) |
| Restaurants, n=2 | ***Advertising & Promotions***<br>• Multiple healthy options advertised in prominent positions including cold breakfast bar, fresh fruit and steamed potatoes (2)<br>• Healthier 'side options' advertised using poster entitled "It's better for you" on the service counter (1)<br>• Poster advertising 'now serving [Brand Name] (no added sugar) in our restaurant' (1)<br>• Poster advertising 100% fruit smoothies (2)<br>• Poster advertising 'lighter breakfast' options including cereal, yoghurt, fruit, mixed nuts (1) | ***Advertising & Promotions***<br>• Price promotion on home-made cookies with self-service tongs at payment areas (1)<br><br>***Layout & prominent placing of healthy & unhealthy items***<br>• Self-service salad bar no longer in use (1)<br>• 'Snack' table in central location near payment areas predominantly serving baked goods (1)<br>• Snack bar and ice-cream freezer located next to payment area and |

For peer review only

| | | |
|---|---|---|
| | ***Layout & prominent placing of healthy & unhealthy items***<br>• First visible stall on entry is a farm stall displaying fresh fruit and vegetables (1)<br>• Choice of 4 packaged salads available in addition to sandwiches (1)<br>• Fruit and healthier snack options displayed near payment area (2)<br>• Healthier and cheaper 'side options' available which include potatoes and vegetable options with clear, comparable prices (2) | cutlery collection area (1)<br>• Menu displayed by restaurant entrance on stall sponsored by Coca-Cola and states 'Coca Cola – complete your meal' (1) |

For peer review only

BMJ Open

# Are food and drink retailers within NHS venues adhering to NICE Quality Standard 94 guidance on childhood obesity? A cross-sectional study of two large secondary care NHS hospitals in England



| | |
|---|---|
| Journal: | *BMJ Open* |
| Manuscript ID | bmjopen-2017-018214.R1 |
| Article Type: | Research |
| Date Submitted by the Author: | 20-Sep-2017 |
| Complete List of Authors: | James, Alice ; University of Bristol, 5th year Medical Student, Faculty of Health Sciences<br>Birch, Laura; University of Bristol, NIHR Bristol Biomedical Research Centre (Nutrition Theme)<br>Fletcher, Peter; Cheltenham General Hospital, Department of General and Old Age Medicine<br>Pearson, Sally; Gloucestershire Hospitals NHS Foundation Trust, Department of Clinical Strategy<br>Boyce, Catherine; Gloucestershire Hospitals NHS Foundation Trust, Department of Clinical Strategy<br>Ness, Andy; University of Bristol, NIHR Bristol Biomedical Research Centre (Nutrition Theme)<br>Shield, Julian; University of Bristol, NIHR Bristol Biomedical Research Centre (Nutrition Theme)<br>Lithander, Fiona; University of Bristol, NIHR Bristol Biomedical Research Centre (Nutrition Theme) |
| <b>Primary Subject Heading</b>: | Paediatrics |
| Secondary Subject Heading: | Nutrition and metabolism, Public health, Health policy |
| Keywords: | PAEDIATRICS, NUTRITION & DIETETICS, Paediatric endocrinology < PAEDIATRICS |
| | |



SCHOLARONE™
Manuscripts

# Are food and drink retailers within NHS venues adhering to NICE Quality Standard 94 guidance on childhood obesity? A cross-sectional study of two large secondary care NHS hospitals in England

List of authors:

A James[1], L Birch[2], P Fletcher[3], S Pearson[4], C Boyce[5], AR Ness[2], J Hamilton-Shield[2], FE Lithander[2]

Corresponding author: Fiona Lithander, fiona.lithander@bristol.ac.uk

Keywords: childhood, obesity, NICE Quality Standard 94, retailers, food, drink

For peer review only

**Abstract**

**Objective**

To assess whether the food and drink retail outlets in two major National Health Service (NHS) district general hospitals in England adhere to Quality Statements 1-3 of the UK National Institute for Health and Care Excellence (NICE) Quality Standard 94.

**Design**

Cross-sectional, descriptive study to assess the food and drink options available in vending machines, restaurants, cafes and shops in two secondary care hospitals.

**Main outcome measures**

Adherence to Quality Statement 1 whereby the food and drink items available in the vending machines were classified as either healthy or less healthy using the Nutrient Profiling Model (NPM). Compliance with Quality Statements 2 and 3 was assessed through the measurement of how clearly the shops, cafes and restaurants displayed nutrition information on menus, and the availability and prominent display of healthy food and drink options in retail outlets, respectively.

**Results**

Adherence to Quality Statement 1 was poor. Of the 18 vending machines assessed, only 7 (39%) served both a healthy food and a healthy drink option. Neither hospital was compliant with Quality Statement 2 wherein nutritional information was not available on menus of food providers in either hospital. There was inconsistent compliance with Quality Standard 3 whereby healthy food and drink options were prominently displayed in the two main hospital restaurants, but all shops and cafes prioritised the display of unhealthy items.

**Conclusions**

Neither hospital was consistently compliant with Quality Statements 1-3 of the NICE Quality Standard 94. Improving the availability of healthy foods and drinks whilst reducing the display and accessibility to less healthy options in NHS venues may improve family awareness of healthy alternatives. Making it easier for parents to direct their children to healthier choices is an ostensibly central component of our health care system.

**Strengths and limitations of this study**

- This is the first study to evaluate the consumer nutrition environment of two NHS hospitals in England, and to assess their compliance to recommendations of the NICE Quality Standards 94.
- Only two hospitals in the same geographical area in England were assessed.
- The inclusion of a second assessor to provide an additional independent evaluation would have been beneficial to reduce possible bias.

For peer review only

**Introduction**

Childhood obesity is a global health concern.[1] Almost 10% of 4-5 year olds and 20% of 10-11 year olds in the UK are currently classified as obese[2, 3] which is known to increase morbidity and mortality in later life through its association with cancer, cardiovascular disease and mental health disorders.[3, 4] The doubling of obesity rates between the first and final years of primary school highlights early childhood as a high-risk period for obesogenic factors to take effect.[2, 3] Whilst overweight and obese children are more likely to become obese adults,[4] healthy weight adults who were obese during childhood have similar risk for metabolic diseases as those who maintained a healthy weight throughout life,[5] suggesting that childhood is an opportune time for obesity prevention.

There is increasing evidence that the consumer nutrition environment is an important determinant of dietary behaviour and obesity.[6-8] The consumer nutrition environment is one of four types of food environments and describes what an individual encounters when he or she enters a venue where food is purchased or consumed.[9] The consumer nutrition environment accounts for factors that influence food choice within shops such as availability, price, promotions, placement, variety, quality and nutrition information of food and drink[7, 10] and it is understood that consumers' dietary choices are affected by these factors.[7, 11]

The UK National Health Service (NHS) recognises that venues such as hospitals are consumer nutrition environments given that hospitals are venues where food is purchased and consumed. Indeed the NICE Quality Standard 94 (Table 1), which is entitled 'Obesity in children and young people: prevention and lifestyle weight management programmes', [4] identifies NHS venues as important settings in which to implement childhood obesity prevention strategies. The NICE Quality Standard 94 specifically refers to the availability of healthy foods and drinks in vending machines, nutrition labelling of menus and the prominently placement of healthy food and drink options in NHS venues.[4] Children may attend an NHS venue, including a hospital, as a patient or a visitor, and can be directly or indirectly influenced by the consumer nutrition environment within a hospital.

Studies conducted in Canada and the USA have assessed consumer nutrition environments in hospitals and have shown that food and drink retailers in both adult[12, 13] and children's[14, 15] hospitals offer unhealthy food and drink options. One of these studies[15] used a tool to assess the consumer nutrition environment by evaluating individual factors such as amount of nutritious food sold at cafeterias, the presence of fast food outlets, the amount of nutritious food alternatives, and the availability of exercise programmes. The other three studies [12-14] used tools which allowed the creation of a composite score of the environment such as the Hospital Nutrition Environment Scan,[12, 13] or the Nutritional Environmental Measured Survey-cafeteria (NEMS-C).[14] There are many other consumer nutrition environment tools in the

For peer review only

literature that have been used in different venues which assess availability, price, variety and quality of fruit and vegetables, advertising, product placement, price promotions and labelling.[7, 16, 17] Regardless of whether these tools assess individual factors or allow the creation of a composite score, it is noteworthy that few have been deemed reliable or valid.[7] Moreover, most tools have been developed and used in the United States and Canada and have not been used outside North America.[7]

Not only is little known about the consumer nutrition environment in NHS Hospitals in the UK but no tool exists which could comprehensively assess it. The current study addresses a gap in the literature by developing a consumer nutrition environment tool to measure the healthfulness of food outlets of NHS hospitals, and assesses this against NICE Quality Standard 94 which refers to the availability of healthy foods and drinks in vending machines, nutrition labelling of menus, and the prominent placement of healthy food and drink options.

For peer review only

Table 1: NICE Quality Standard 94 “Obesity in children and young people: prevention and lifestyle weight management programmes”

| | Quality Statement | How it was assessed and how compliance was defined |
|---|---|---|
| Quality Statement 1 | Children and young people, and their parents or carers, using vending machines in local authority and NHS venues can buy healthy food and drink options | Foods and drinks were classified as healthy or less healthy using the NPM. A hospital was deemed compliant if it housed one vending machine which contained both one healthy food and one healthy drink |
| Quality Statement 2 | Children and young people, and their parents or carers, see details of nutritional information on menus at local authority and NHS venues. | A hospital was deemed compliant if the majority of its retails outlets displayed nutritional information on their menus |
| Quality Statement 3 | Children and young people, and their parents or carers, see healthy food and drink choices displayed prominently in local authority and NHS venues | Foods and drinks were defined as healthy if they were within the four food groups in the Eatwell Guide. A hospital was deemed compliant if the majority of its retail outlets prominently displayed healthy food and drinks options |

NPM, nutrient profile model

For peer review only

**Methods**

*Setting/Hospitals*

This study was conducted in two major District General Hospitals in the South West of England in July 2016. These two NHS hospitals were chosen as the lead author was studying in these hospitals during this period. Both hospitals offer adult inpatient care; paediatric, day-time access for assessment and out-patients are available in both, but only the larger hospital, provides paediatric inpatient care. One Hospital has approximately 680 beds in total, 8 vending machines and a total of 6 food retail outlets which comprise 1 restaurant, 3 cafes and 2 shops. The other has approximately 380 beds, 10 vending machines and 7 food retail outlets comprising 1 restaurant, 2 cafes and 3 shops. The two restaurants served hot and cold meals throughout the day. All shops sold sweet and savoury snack items, cold drinks and pre-packaged sandwiches and salads. The only meals served by the cafes were sandwiches and salads in addition to confectionary, savoury snack items, and hot drinks. All outlets were accessible to both staff and patients. The only two outlets which appeared to differentially target staff rather than patients were the two cafes, one on each hospital site, within the respective Education Centres where staff training and meetings take place, and where several members of staff hold offices. Food and drink retailers in each hospital were evaluated against Quality Statements 1-3 of NICE Quality Standard 94 (Table 1). Data were collected by the lead author during a 2 week period in July 2016 where each vending machine and retail outlet was visited by her on one occasion only.

*Assessment Tool*

The Consumer Nutrition Environment Tool (C-NET) was developed by the lead author and used two different methods to measure adherence to Quality Standards 1-3 within NICE Quality Statement 94.

*Quality Statement 1: "Children and young people, and their parents or carers, using vending machines in local authority and NHS venues can buy healthy food and drink options".*

To measure adherence to Quality Statement 1, the user recorded each food and beverage item for sale in each of the vending machines. The nutrient composition for each item was then retrieved from the item packaging or from product nutrition information available online. The UK Department of Health Nutrient Profiling Model (NPM) was used to classify these foods and drinks into two categories; less healthy and healthy, and details of this model and the scoring system have been published elsewhere.[18] To do this, the nutrient content of each food and beverage was assessed against a set of published criteria to determine whether it contains certain nutrients above or below particular thresholds. The NPM identifies foods and drinks that are high in fat, salt or sugar and enables them to be differentiated into two categories, healthy or less healthy, based on their nutrient composition. The NPM uses a scoring system where points are allocated

For peer review only - http://bmjopen.bmj.com/site/about/guidelines.xhtml
For peer review only

on the basis of the nutrient content of 100g of a food or beverage. Points are awarded for energy, saturated fat, total sugar and sodium ('A' nutrients) and fruit, vegetable and nut content, fibre and protein ('C' nutrients). The score for 'C' nutrients is subtracted from the 'A' nutrients score to give a final NPM score. Foods that score four points or more, and drinks which score one or more points are classified as 'less healthy' using the NPM. An example is that of raw nuts which had a nutrient composition (per 100g) as follows: 2656kJ, 9.1g saturated fat, 3.7g total sugar, 0.02g salt; this item scored 16 'A' points. Upon calculation of 'C' points for the raw nuts (per 100g), they contained >80% fruit, vegetables or nuts, 6g fibre, and 15.8 g protein, giving it a total of 5 'C' points. The final score for this item was 1, hence this item was classified as healthy according to the NPM. The number of vending machines within each NHS venue where each food and beverage was available was recorded and data collected were used to measure compliance with Quality Statement 1. The NPM was developed by the Food Standards Agency in 2004-2005, and was subject to rigorous scientific scrutiny, extensive consultation, and review. It is supported by the independent Scientific Advisory Committee on Nutrition and a wide range of nutrition experts.[19] The NPM was introduced as mandatory in 2007 by the UK Office of Communications and DH to regulate food and drinks in the context of television advertising to children.

*Quality Statements 2 and 3, respectively: "Children and young people, and their parents or carers, see details of nutritional information on menus at local authority and NHS venues" and "Children and young people, and their parents or carers, see healthy food and drink choices displayed prominently in local authority and NHS venues".*

The NPM was not used to measure compliance with Quality Statements 2 or 3; this was done using a more subjective assessment of the consumer nutrition environment by the lead author. To measure adherence to Quality Statements 2 and 3, she evaluated the quality of the consumer nutrition environment within each hospital restaurant, café or shop through answering a series of yes/no questions which centred around 3 main themes: the provision of nutrient information for meals on menus (Quality Statement 2), advertising and promotions (Quality Statement 3) and the prominent placing of healthy and unhealthy items in the retail outlets (Quality Statement 3) as outlined in Table 2. The themes were derived directly from the two Quality Statements. For assessment against Quality Statement 2, for example, the lead author examined the retail outlets for the display of calories, fat, saturated fat and sugar on menus, as specified in the Quality Statement itself. Quality Statement 2 was not relevant to the shops as they did not have menus.

For peer review only

Table 2**:** Consumer Nutrition Environment Tool (C-NET); the questions were asked to ascertain if retail outlets adhered to Quality Statements 2 and 3. Questions around nutritional information for hot and cold meals relate to Quality Statement 2. Questions around advertising and promotions, and the layout & prominent placing of healthy and unhealthy items relate to Quality Statement 3.

***Nutritional information for hot and cold meals.*** *These questions relate to Quality Statement 2 "Children and young people, and their parents or carers, see details of nutritional information on menus at local authority and NHS venues"*
Are comprehensive details on nutritional content available for sandwiches, salads and other cold and packaged meal options?
If this information is not provided, are the ingredients listed?
Are comprehensive details on nutritional content available for hot meal options?
If this information is not provided, are details provided about the ingredients and cooking methods?
Are posters or labels used to direct consumers to healthier meal options?

***Advertising & Promotions.*** *These questions relate to Quality Statement 3 "Children and young people, and their parents or carers, see healthy food and drink choices displayed prominently in local authority and NHS venues".*
Are signs or other promotional materials used to advertise healthy options?
Are signs or other promotional materials used to promote unhealthy options?
Are there advertisements for unhealthy items that are clearly aimed at children? For example the use of bright colours, television/cartoon characters and/or specific wording of advertisement?
Are price incentives (e.g. reduced price offers) for healthy items clearly displayed (e.g. using signs)?
Are price incentives (e.g. reduced price offers) for unhealthy items clearly displayed (e.g. displayed at payment area)?

***Layout & prominent placing of healthy & unhealthy items.*** *These questions relate to Quality Statement 3 "Children and young people, and their parents or carers, see healthy food and drink choices displayed prominently in local authority and NHS venues".*
Are healthy food and drink items promoted via prominent placing e.g. near the entrance to a shop or as part of a large/attractive display?
Are unhealthy food & drink options promoted via prominent placing?
Are unhealthy options displayed at the payment area or point of purchase?
Are unhealthy options displayed at an easy height for children to 'grab' (1 metre height)

For peer review only

For Quality Statement 3, the lead author's knowledge of the Eatwell Guide[20] was used whereby foods within the food groups such as fruit, vegetables, dairy, wholegrain and high fibre foods, eggs, lean meat and pulses, were classified as healthy, and foods and drinks which are high in fat, salt and sugars such as biscuits, sweets, chocolate, and sugar sweetened beverages were classified as unhealthy. Quality Statement 3 includes phrases such as 'can easily find' healthy food, and that these items were 'prominently displayed' and descriptions of these terms are outlined in Table 2.

*Procedure*

Compliance with Quality Statement 1 was observed if a NHS venue had a vending machine where at least one healthy food and at least one healthy beverage option were available for sale in the same machine. Compliance with Quality Statement 2 was observed if retail outlets provided information on energy, total fat, saturated fat, salt and sugar content of meals and snacks. Quality Statement 2 states that listing ingredients and cooking methods constitutes an acceptable level of nutritional information if the information on energy, fat, saturated fat, salt and sugar content is not available. Drinks were not assessed as part of Quality Statement 2 since the rationale provided by NICE refers to food only. When nutrient information was not clearly displayed, the lead author liaised with members of catering staff to identify whether this information would be available on request. With respect to Quality Statement 3, the lead author examined whether healthy and less healthy foods and drinks were advertised outside the venues, whether advertising stalls containing food and drink items were placed nearest the door of the shop, café or restaurant, whether items were at a child's eye level which was classified as 1 metre height, and whether items were displayed at the payment area. Compliance with Quality Statement 3 was observed if healthy food and drink choices were displayed prominently in the retail outlets and poor compliance was deemed if unhealthy options were prominently displayed. Items were deemed to be prominently displayed if they were placed at the entrance to the retail outlet and hence visible to those walking past, beside queuing and payment areas, or if they were accompanied by signs advertising the product or detailing price promotions. Though price was not specifically mentioned in Quality Statements 1, 2 or 3, the price of vending machine items and the existence of price promotions were both assessed as part of this study. Quality Statement 1 asks that venues ensure that children and their carers 'can buy' healthy items and Quality Statement 3 requires that healthy food and drink items are 'promoted'. Both Statements thus require reasonable accessibility to healthy options. Accessibility applies not only to the physical display and number of items, but also to the financial accessibility. The definition of compliance with the three Quality Statements was selected by the authors given the lack of guidance regarding what constituted compliance provided by NICE Quality Standard 94.

*Data analyses*

For peer review only

217 Data on the NPM scored are presented as mean (sd) and range. Qualitative data were initially coded and collated into themes by the lead author. Interpretation of these data and the identification of themes was reviewed and discussed by multiple authors (AJ, LB, FEL) throughout the process.

For peer review only

## Results

Two restaurants, 5 cafes, 5 shops and 18 vending machines were included in the analyses across both hospital sites. Data were originally collected from 29 vending machines but 11 were excluded from analyses. Ten vending machines, which sold hot drinks only, were excluded because nutrition information was not clearly displayed nor was it available online or from the supplier when requested by the lead author. One vending machine, which sold only frozen items such as burgers, was also excluded because this machine was removed from the hospital before the end of the data collection period.

*Quality Statement 1: "Children and young people, and their parents or carers, using vending machines in local authority and NHS venues can buy healthy food and drink options"*

Of the 18 vending machines, 7 (39%) offered both a healthy food and a healthy drink (Table 3). 55 different items were on sale across all 18 vending machines, 40 of which were foods and 15 were drinks. Where the same drink was served in a 330ml can or in a larger 500ml bottle, this was classified as two separate drinks. When the 40 foods alone were assessed using the NPM, the mean (sd) NP score was 18.3, (9.3). Only 4 of the 40 (10%) foods were classified as healthy with a NP score of less than 4; these items were baked crisps, a packet of dried fruit and nuts, a packet of raw nuts and a muesli bar (mean (sd) NP score -1.3 (4.5)). When the other 36 (90%) food items were analysed according to the NPM, the mean (sd) NP score was 20.5 (6.8), significantly greater than the cut-off for the less healthy classification of 4 points or more. The 4 healthy foods items were priced equivalently to similar less healthy items available in the vending machines.

When the drinks were assessed, 8 of the 15 (53%) available were classified as healthy (NP score -0.5 (sd 1.4)) using the NPM cut-off of 1 point or more, and the remainder classified as less healthy (NP score 1.7 sd (0.5)). The drinks that were classified as healthy were an orange flavoured sugar sweetened beverage, concentrated orange juice, bottled water, and sugar-free cola drinks.

For peer review only

Table 3: Mean (sd) nutrient profile model scores for all 40 foods and 15 drinks found in the vending machines; data includes healthy and less healthy foods and drinks. A food is classified as less healthy where it scores ≥4 points. A drink is classified as less healthy where it scores ≥ 1 point

| | Mean | sd | Range |
|---|---|---|---|
| Crisps, n=10 | 10.3 | 4.3 | 1, 17 |
| Chocolate, n=19 | 25.6 | 1.3 | 23, 27 |
| Sweets, n=2 | 15.5 | 0.7 | 15, 16 |
| Sweet & savoury biscuits, n=4 | 19.8 | 6.7 | 10, 25 |
| Dried fruit and / or nuts, n=5 | 6.4 | 12.8 | -8, 25 |
| Drinks, n=15 | 0.5 | 1.6 | -4, 2 |

*Quality Statement 2: "Children and young people, and their parents or carers, see details of nutritional information on menus at local authority and NHS venues"*

Neither hospital was compliant with Quality Statement 2. When the two restaurants were analysed against Quality Statement 2, it was found that neither displayed nutrition information on their menus for hot or cold meals. When the lead author asked the retail staff, she was informed that this information was available only for cold fillings offered with jacket potatoes and for a proportion of the filled sandwiches on sale. Quality Statement 2 states that if the nutrient content of a recipe is unavailable to consumers, the ingredients and cooking methods should be available. Whilst this information was not displayed on menus, catering staff advised that this information would be available on request, though it was not specifically requested by the lead author as part of this study. Though not displayed on menus, a range of sandwiches and salads that were made onsite were available in the restaurants. Nutritional information detailing total energy, fat, saturated fat, sugar and salt content was displayed on the labels of these items.

None of the cafes provided nutrient information on their menus, meaning that all five cafes were not compliant with Quality Statement 2. However, three of the cafes offered pre-packaged sandwiches and comprehensive nutrition information was available on the packaging of these. The remaining two cafes sold sandwiches, baguettes and salads made by a local catering company. Catering staff in these cafes advised that specific nutrition information was not available for these items but that details of ingredients were available on request, though the lead author did not specifically request this information.

For peer review only

*Quality Statement 3: "Children and young people, and their parents or carers, see healthy food and drink choices displayed prominently in local authority and NHS venues".*

There was inconsistent compliance to Quality Statement 3 in both hospitals (Table 4). When the two restaurants were analysed, adherence to Quality Statement 3 varied. One of the restaurants housed a stall inside the entrance, which sold fresh fruit, vegetables, and local produce and was labelled as a 'Farm Shop'. The same restaurant also sold steamed potatoes and these, in addition to fresh vegetables, were clearly advertised as a cheaper option than a portion of chips. In the second restaurant, there were several signs which advertised 'Healthier Options'. This included a 'lighter breakfast' which comprised grapefruit segments, natural yoghurt, a bowl of breakfast cereal with milk, a pastry and a cheese portion, both of which are less healthy options. Healthier snack options such as fresh fruit were prominently displayed on service counters and by the payment area. All five cafes demonstrated good adherence to Quality Statement 3. The balance of healthy and less healthy items on display in the most prominent areas such as the queuing or payment area was equal and there was less evidence of advertising and price promotions on less healthy items than in the shops. Fresh fruit was available beside the payment areas in 4 of the 5 cafes. However baked goods such as muffins, scones, cookies and cakes were displayed in glass cabinets or on the counter surface next to the queuing and payment area in all 5 cafes.

For peer review only

Table 4. Observed activities which shops, cafes and restaurants engaged in against which compliance to Quality Statement 3 was measured. The number in parentheses is number of shops or cafes or restaurants that engaged in this activity.

<table>
<tr><td></td><td colspan="2">Quality Statement 3: “Children and young people, and their parents or carers, see healthy food and drink choices displayed prominently in local authority and NHS venues”.</td></tr>
<tr><td></td><td>Compliant</td><td>Non-compliant</td></tr>
<tr><td>Shops, n=5</td><td>Advertising & Promotions<br>• ‘Meal-deal’ promotion includes sugar-free drink options (5)<br>• Poster advertising price promotions on fresh fruit and dried fruit (2)</td><td>Advertising & Promotions<br>• Fruit available was not reflective of the fruit advertised on the poster (2)<br>• ‘Big Kids Sweet Zone’ offering 35 different products at ‘pocket money’ prices (1)<br>• Posters advertising price promotions on unhealthy options including share-size confectionary, sugar-sweetened beverages, crisps, ‘coffee & muffin deal’ hotdogs and ice-cream (5)<br>• ‘Low saturated fat’ sandwich range available but not advertised (2)<br>• ‘Meal-deal’ poster advertising sandwich, crisps or piece of fruit plus a drink although the fruit option was unavailable in some shops (3)<br><br>Layout & prominent placing of healthy & unhealthy items<br>• Fresh fruit stall located at the back of the shop (1)<br>• Bruised fruit (1)<br>• First visible items on entry were pic’n’mix stalls, confectionary and crisps (5)<br>• Floor-to-ceiling confectionary stall adjacent to the entrance (1)<br>• Multiple rows of confectionary at 1m height (5)<br>• Ice-cream freezer at the entrance and visible from main hospital concourse (2)<br>• Homemade cakes prominently displayed at payment areas (2)<br>• Price promotions on unhealthy snack items prominently displayed at payment areas (2)</td></tr>
</table>

For peer review only

| | | |
|---|---|---|
| Cafes, n=5 | ***Advertising & Promotions***<br>• Poster advertising healthier wraps (roast chicken rainbow/sweet potato/falafel) (1)<br>• Skimmed/semi-skimmed/soya milk advertised as options for drinks & porridge (1)<br>• Poster and table-top advertisements for fruit smoothies containing 100% fruit in 500ml portions stating '1 of your 5 a-day' (1)<br><br>***Layout & prominent placing of healthy & unhealthy items***<br>• 'Healthier range' wraps/sandwiches/salads available and clearly labelled (1)<br>• Healthy breakfast options clearly displayed. Options included porridge pots, fruit salad and yoghurt (2)<br>• Fresh fruit salad tubs prominently displayed in glass display cabinet (1)<br>• Salads served without dressing. Self-service dressings available (1)<br>• Prices available for soup with or without a bread roll (1)<br>• 2 healthier snack options prominently displayed which were cereal bars and healthier biscuits (2)<br>• Fresh fruit prominently displayed although no price information was available (1)<br>• Baked crisps available (2) | ***Advertising & Promotions***<br>• Price promotions on baked goods such as brownies displayed prominently next to the payment area (1)<br>• Poster advertisement in main hospital foyer outside café for 'double chocolate cookie mocha creamy cooler' (1)<br>• Poster advertisement for 'Product of the month' which were cookies (1)<br><br>***Layout & prominent placing of healthy & unhealthy items***<br>• Cakes and other baked goods prominently displayed at service and payment areas (5)<br>• No healthy snacks or fruit available (1)<br>• Crisps and salted nuts prominently displayed next to café entrance and in front of healthier snack items (2)<br>• Large chocolate bar selection available (5) |
| Restaurants, n=2 | ***Advertising & Promotions***<br>• Multiple healthy options advertised in prominent positions including cold breakfast bar, fresh fruit and steamed potatoes (2)<br>• Healthier 'side options' advertised using poster entitled "It's better for you" on the service counter (1)<br>• Poster advertising 'now serving (Brand Name) (no added sugar) in our restaurant' (1)<br>• Poster advertising 100% fruit smoothies (2)<br>• Poster advertising 'lighter breakfast' options including cereal, yoghurt, fruit, mixed nuts (1)<br><br>***Layout & prominent placing of healthy & unhealthy items*** | ***Advertising & Promotions***<br>• Price promotion on home-made cookies with self-service tongs at payment areas (1)<br><br>***Layout & prominent placing of healthy & unhealthy items***<br>• Self-service salad bar no longer in use (1)<br>• 'Snack' table in central location near payment areas predominantly serving baked goods (1)<br>• Snack bar and ice-cream freezer located next to payment area and cutlery collection area (1)<br>• Menu displayed by restaurant entrance on stall sponsored by Coca-Cola and states 'Coca Cola – complete your meal' (1) |

For peer review only

| | | |
|---|---|---|
| | • First visible stall on entry is a farm stall displaying fresh fruit and vegetables (1)<br>• Choice of 4 packaged salads available in addition to sandwiches (1)<br>• Fruit and healthier snack options displayed near payment area (2)<br>• Healthier and cheaper 'side options' available which include potatoes and vegetable options with clear, comparable prices (2) | |

For peer review only

Healthy options made up approximately 25% of the food and beverage items available in the shops and these items were displayed in less prominent locations such as at the back of the shop or on low-lying shelves. Posters were used in all of the hospital shops and cafes to advertise price promotions on less healthy options including signs placed outside shops and cafes to advertise items such as hot dogs and ice cream. Cakes made onsite were displayed next to the payment area in 2 of the shops, and less healthy options including muffins and chocolate bars were displayed at the payment areas in all shops and cafes. Price promotions were advertised for several of these less healthy products displayed at the payment area.

The layout of the unhealthy items in many of the shops appeared to target children. The 'pic n mix' sweet stalls in both of the largest shops had prominent positions near the entrance and products such as colourful sugar-sweetened iced drinks, lollipops and chocolate eggs containing small toys were displayed at the payment area or at a child's eye level. There was a display in one shop with a sign which read 'Big Kids Sweet Zone' offering 35 different sweets all priced at less than £1. Both of the largest shops used posters to advertise a price deal on fresh fruit, however fruit stalls were not in a prominent location and were poorly stocked.

For peer review only

## Discussion

This study is the first to describe the consumer nutrition environment of two NHS hospitals in England and to assess their compliance with NICE Quality Standard 94. We found that food and drink retailers in these two hospitals demonstrated poor compliance with this Quality Standard. Only 39% of vending machines across both hospitals served both one healthy food and one healthy drink option indicating poor compliance with Quality Statement 1. Moreover, 90% (36/40) of the food items in the vending machines were classified as less healthy suggesting that the consumer may have difficulty identifying and locating the 10% of items classified as healthy. 53% (8/15) of the drink items available were classified as unhealthy. It was found that the two hospitals were not compliant with Quality Statement 2, which refers to the availability of nutritional information at the point of choosing food or drink. Compliance was variable in relation to Quality Statement 3, where restaurants engaged in various activities in the display of healthy options yet all cafes and shops favoured the prominent display and advertising of unhealthy foods and drinks.

Hospitals have a role to play in advocating for healthy lifestyle and good nutrition.[21, 22] Choice architecture describes the concept that behaviour could be changed in anticipated ways by changing the environments where people make choices.[23, 24] The alteration of micro-environments, which are settings where people may congregate for a specific purpose,[25] including hospitals, may be one approach to encourage healthier dietary behaviour, and studies have shown that minor changes in accessibility to food can decrease food intake.[26] The consumer nutrition environment in 14 children's hospitals in California was assessed and it has been suggested that nutrition intervention is needed to improve the availability of healthy food and beverage options.[14] The authors of that research suggest that inexpensive interventions could be used such as providing nutrient information and introducing signage that promotes healthy choices.[14] McDonald and co-workers[15] reported that university-affiliated children's hospitals in Canada and the US provide a suboptimal health environment, and hypothesise that a reliance on revenue from outlets which provide less nutritious foods may be a factor. Such results are not restricted to children's hospitals however. Winston and co-workers[12, 13] described the nutrition environment of 39 hospitals in the USA, and found that the consumer nutrition environment was poor and suggested that dietary interventions are justified in health settings.

The current study found that only 39% of vending machines provided both a healthy food and a healthy drink, and yet this result must be interpreted with caution. The most widely available healthy food item was baked crisps which were found in 33% vending machines, and when presented in the same vending machine as a healthy drink such as bottled water or a sugar-free drink, the machine, and by extension the hospital, was classified as compliant with Quality Statement 1. It is known that nutrient profile schemes have become drivers for product reformulation[18] and the baked crisps, and indeed the orange flavoured sugar sweetened

For peer review only

beverage which was classified as healthy, may have been reformulated to meet the NPM criteria. It is noteworthy that both of these items are classified as unhealthy according to the Eatwell Guide[20] where they are both in the category of foods which are high in fat, salt and sugar yet are classified as healthy when the NPM was used.

It is well accepted that nutrition information at the point of purchase can influence food choice.[27] Consumers under-estimate by 2 to 4 times the saturated fat, energy and sodium content of restaurant foods,[27] yet providing accurate point-of-sale nutrition information is known to improve consumer choice.[27] None of the restaurants, cafes or shops examined in the current study were compliant with Quality Statement 2, which states that nutrition information should be available for consumers at the point of choosing food and drink options. It is a hospital's duty to empower consumers with the information required to make an informed choice.[28] When asked, staff suggested that nutrition information would be provided to consumers on request yet the format of this information, exactly when it would be provided, and how user-friendly it would be, is unknown.

The availability and accessibility of unhealthy foods have been identified as risk factors for overeating[29] and it is understood that the prominence of food and drinks displays can influence consumer choice.[30, 31] In the current study, the restaurants demonstrated variable compliance to Quality Statement 3 which states that healthy food and drink options are displayed prominently. Evidence of good practice included one restaurant which advertised fresh fruit and vegetables at competitive prices, whilst the other advertised what was referred to as a 'lighter breakfast'. This option, however, also included a pastry and cheese portion, both of which are known to be high in fat, saturated fat and salt. The 5 shops and 5 cafes all prioritised the prominent display of unhealthy options, and whilst fruit was available beside the payment area in 4 of the 5 cafes albeit poorly stocked, so too were unhealthy baked goods which were displayed by the payment area in all 5 cafes. Research has shown that unhealthy food and drink items are difficult for consumers to avoid in supermarkets, as not only do they take up more shelf space than healthy items such as fruit and vegetables, but are more often displayed at payment areas, as seen in the current study.[32-34] Moreover, the placement of healthy food items at a payment area can lead to a substantial positive impact on sales of these products.[35] In addition, both of the largest shops in the current study displayed an array of sweets, chocolate and sugar-sweetened drinks at child's eye level, and evidence suggests that placing products on shelves at eye-level positively influences sales.[30]

The Consumer Nutrition Environment Tool (C-NET) used in the current study was developed by the lead author of this study. Whilst this tool has not been validated, it allowed both the objective collection of data

For peer review only

using the NPM, and the subjective assessment of marketing practices that contribute to food purchases using a series of questions (Table 2). Other studies which have assessed food retailers in hospitals have used a variety of methods; some relied on telephone interviews with cafeteria directors which may have biased the data collected[15] whilst others used ~~a validated~~ tool such as the University of Pennsylvania Nutrition Environment Measures Scale (NEMS). However, no equivalent British tool was found and thus the lead author developed the C-NET. This model was selected for use in the current study as it is widely regarded as scientifically robust and effective in identifying less healthy items, and in practical terms, it is well established in the UK. There are other government endorsed NPMs available, such as the EU Pledge model and the WHO Europe model, but these use multiple categories and subcategories of foods which have raised concerns of ambiguity and additional complexity that may reduce the clarity and transparency of the models.[36] They also do not have the same track record of effective use in the UK regulatory environment. The Committee of Advertising Practice (CAP), the lead UK organisation that write and maintain the UK Advertising Codes to ensure advertising in the UK is legal, decent, honest and truthful, have adopted the DH NPM in their recently published regulatory statement on food and soft drink advertising to children, which was developed following public consultation and will come into effect in the UK on 1 July 2017.[37]

Consumer organisations have set up campaigns in the UK and Australia asking supermarkets to remove unhealthy food and drink items from payment and queuing areas[38-40] but it is not known if such campaigns exist for retails outlets in hospitals. NICE Quality Standards are a set of prioritised statements, which draw on existing guidance to set out the priority areas for quality improvement in health and social care. They are designed to drive measurable quality improvements yet, using them in the current study has proven challenging. Quality Statement 1 refers to the availability of ‘healthy food and drink options’ in vending machines, yet nowhere in NICE Quality Standard 94 or in the associated documents is there a clear definition of what ‘healthy’ means, and it was on this basis that the DH NPM was chosen as a method of classification. Moreover, the definition of compliance to Quality Statement 1, where a vending machine contained both a healthy food and a healthy drink was chosen by the study authors given the lack of guidance on what constitutes as compliance from NICE Quality Standard 94. The authors considered compliance to Quality Statements 2 and 3 if most retail outlets in the hospitals adhered to the respective statement. ‘Evidence’ of ‘arrangements to display healthy food and drink options in prominent places’ constitutes adherence to Quality Statement 3 according to NICE Quality Standard 94. However, challenges arose in assessing what constituted a ‘healthy option’ and a ‘prominent place’, and was subjective. The measurement of adherence to all three Quality Statements is open to interpretation, and assessment of adherence was a challenging task given their non-quantifiable and non-specific nature. NICE Quality Standard 94 would benefit from being

For peer review only

For peer review only - http://bmjopen.bmj.com/site/about/guidelines.xhtml

measurable and, in the first instance, hospital venues could strive towards a balance of 50% healthy and 50% less healthy food and drinks.

The main limitation of the current study is that only two hospitals were included, both of which are in the same geographical area in England. Moreover, a second assessor would have provided an additional independent evaluation of the offerings. A further limitation is that the methodological approach for Quality Statements 2 and 3 is qualitative and subjective in nature, and future studies could use published tools which provide quantitative measurement variables and describe a systematic approach to data collection.[7, 41] Further research is needed on a more representative sample of hospitals to fully understand compliance to NICE Quality Standard 94. However, such a study would be subject to similar limitations in terms of the vague nature of this Quality Standard and a more objective means of assessing adherence to the Statements within the Quality Standard would be necessary.

In conclusion, the current study showed that two NHS hospitals demonstrated poor compliance with Quality Statements 1-3 in NICE Quality Standard 94. The lack of availability of healthy foods and drinks, the absence of nutritional information on the menus, the lack of advertising and display of healthy items and the consistent advertising and prominent display of unhealthy items highlights that improvements are required in NHS venues such as hospitals. These findings have been shared with the NHS Trust of one of these hospitals and re-evaluation will take place in the future. However, as the prevalence of childhood obesity continues to rise globally, it is important that every opportunity is taken to improve the nutrition environment for children's food choices. Hospitals have a duty to provide consumers with the information required to make informed nutrition choices and should take the lead in supplying food and drinks that reflect evidence based nutrition.[28]

For peer review only

Author affiliations:
[1]5th year medical student, Faculty of Health Sciences, University of Bristol
[2]National Institute for Health Research Biomedical Research Centre, Bristol (Nutrition Theme), University Hospitals Bristol NHS Foundation Trust and the University of Bristol, UK
[3]Department of General and Old Age Medicine, Cheltenham General Hospital, Gloucestershire, UK,
[4]Department of Clinical Strategy, Gloucestershire Hospitals NHS Foundation Trust, Gloucestershire, UK
[5]Department of Clinical Strategy, Gloucestershire Hospitals NHS Foundation Trust, Gloucestershire, UK

Acknowledgements

The authors thank Amanda Chong for assistance in the preparation of the manuscript and acknowledge support from the National Institute of Health Research (NIHR) Bristol Nutrition Biomedical Research Unit.

Contributors

AJ and JHS designed and collected the data. PF, SP and CB supervised and oversaw AJ during the data collection period. LB calculated and analysed the nutrient model scores. FEL, LB and ARN supervised the data analyses and prepared the manuscript. All authors revised the manuscript and approved the final version.

Funding

This research received support from the from the National Institute of Health Research (NIHR) Bristol Nutrition Biomedical Research Unit.

Data sharing

No additional data are available.

Competing interests

No competing interests.

For peer review only

## References


1. Lobstein T, Jackson-Leach R, Moodie ML, *et al.* Child and adolescent obesity: part of a bigger picture. *Lancet*. 2015 Jun 20;385(9986):2510-20.
2. NHS Digital. *National Child Measurement Programme England, 2015/16 school year*. 2016. https://www.gov.uk/government/statistics/national-child-measurement-programme-england-2015-to-2016-school-year (accessed 01 Dec 2016).
3. Public Health England. *About Obesity The National Obesity Observatory*. 2016. http://www.noo.org.uk/NOO_about_obesity (accessed 31 November 2016).
4. National Institute for Health and Care Excellence. *Obesity in children and young people: prevention and lifestyle weight management programmes. Quality Standard 94 (QS94).* 2015. https://www.nice.org.uk/guidance/qs94 (accessed 31 Oct 2016).
5. Juonala M, Magnussen CG, Berenson GS, *et al.* Childhood adiposity, adult adiposity, and cardiovascular risk factors. *N Engl J Med*. 2011;365(20):1876-85.
6. Caspi CE, Sorensen G, Subramanian SV, *et al*. The local food environment and diet: a systematic review. *Health Place*. 2012;18(5):1172-87.
7. Black C, Ntani G, Inskip H, *et al.* Measuring the healthfulness of food retail stores: variations by store type and neighbourhood deprivation. *Int J Behav Nutr Phys Act*. 2014;11:69.
8. Department of Health. *NHS Constitution for England*. 2012. https://www.gov.uk/government/publications/the-nhs-constitution-for-england (accessed 31 Oct 2016).
9. Holsten JE. Obesity and the community food environment: a systematic review. *Public Health Nutr*. 2009;12(3):397-405.
10. Glanz K, Sallis JF, Saelens BE, *et al*. Healthy nutrition environments: concepts and measures. *Am J Health Promot*. 2005;19(5):330-3.
11. Hawkes C. Dietary implications of supermarket development: a global perspective. *Dev Policy Rev*. 2008;26(6):657-92.
12. Winston CP, Sallis JF, Swartz MD, *et al*. Consumer nutrition environments of hospitals: an exploratory analysis using the hospital nutrition environment scan for cafeterias, vending machines, and gift shops, 2012. *Prev Chronic Dis*. 2013;10:E110.
13. Winston CP, Sallis JF, Swartz MD, *et al*. Reliability of the hospital nutrition environment scan for cafeterias, vending machines, and gift shops. *J Acad Nutr Diet*. 2013 ;113(8):1069-75.
14. Lesser LI, Hunnes DE, Reyes P, *et al.* Assessment of food offerings and marketing strategies in the food-service venues at California Children's Hospitals. *Acad Pediatr*. 2012;12(1):62-7.
15. McDonald CM, Karamlou T, Wengle JG,*et al*. Nutrition and exercise environment available to outpatients, visitors, and staff in Children's hospitals in Canada and the United States. *Arch Pediatr Adolesc Med*. 2006;160(9):900-5.
16. Gustafson A, Hankins S, Jilcott S. Measures of the consumer food store environment: a systematic review of the evidence 2000–2011. *J Community Health*. 2012;37(4):897-911.
17. Ohri-Vachaspati P, Leviton LC. Measuring food environments: a guide to available instruments. *Am J Health Promot*. 2010;24(6):410-26.
18. Rayner M, Scarborough P, Lobstein T. *The UK Ofcom Nutrient Profiling Model: Defining 'healthy' and 'unhealthy' foods and drinks for TV advertising to children*. 2009. https://www.ndph.ox.ac.uk/cpnp/about/publications-and-reports/group-reports/uk-ofcom-nutrient-profile-model.pdf (accessed 31 Oct 2016).
19. Department of Health. *The nutrient profiling model*. 2011. https://www.gov.uk/government/publications/the-nutrient-profiling-model (accessed 25 Oct 2016).
20. Public Health England. *The Eatwell Guide 2016*. 2017. https://www.gov.uk/government/publications/the-eatwell-guide (accessed 31 Oct 2016).
21. Trinidad C. McDonald's joins Childrens Hospital of Los Angeles. *Calif Hosp*. 1990;4(1):39-40.
22. Cram P, Nallamothu BK, Fendrick AM, *et al*. Fast food franchises in hospitals. *JAMA*. 2002;287(22):2945-6.


For peer review only

23. Hollands GJ SI, Marteau TM, Jebb SA, Kelly MP, Nakamura R, *et al*. Altering micro-environments to change population health behaviour: towards an evidence base for choice architecture interventions. *BMC Public Health*. 2013;13:1218.

24. Marteau TM, Ogilvie D, Roland M, Suhrcke M, Kelly MP. Judging nudging: can nudging improve population health? . *BMJ*. 2011;342:d228.

25. Swinburn B, Egger G, Raza F. Dissecting obesogenic environments: the development and application of a framework for identifying and prioritizing environmental interventions for obesity. *Prev Med*. 1999;29(6):563-70.

26. Rozin P, Scott S, Dingley M, Urbanek JK, Jiang H, Kaltenbach M. Nudge to nobesity I: minor changes in accessibility decrease food intake. *Judgment Decis Making*. 2011;6(4):323-32.

27. Burton S, Creyer EH, Kees J, *et al*. Attacking the obesity epidemic: the potential health benefits of providing nutrition information in restaurants. *Am J Public Health*. 2006;96(9):1669-75.

28. Freedhoff Y, Stevenson R. Frying up hospital cafeteria food. *CMAJ*. 2008;179(3)213-4.

29. Brownell K. Does a "toxic" environment make obesity inevitable? *Obes Manag*. 2005;1(2):52-5.

30. Chandon P, Hutchinson JW, Bradlow E, *et al*. Does in-store marketing work? Effects of the number and position of shelf facings on brand attention and evaluation at the point of purchase. *J Marketing*. 2009;73(6):1-17.

31. Inman JJ, Winer RS, Ferraro R. The interplay among category characteristics, customer characteristics, and customer activities on in-store decision making. *J Mark.* 2009;73(5):19-29.

32. Farley T, Rice J, Bodor JN, *et al*. Measuring the food environment: shelf space of fruits, vegetables, and snack foods in stores. *J Urban Health*. 2009;86(5):672-82.

33. Gebauer H, Laska MN. Convenience stores surrounding urban schools: an assessment of healthy food availability, advertising, and product placement. *J Urban Health*. 2011;88(4):616-22.

34. Thornton LE, Cameron AJ, McNaughton SA, *et al.* Does the availability of snack foods in supermarkets vary internationally? *Int J Behav Nutr Phys Act*. 2013;10(56).

35. Sigurdsson V, Larsen NM, Gunnarsson D. Healthy food products at the point of purchase: an in-store experimental analysis. *J Appl Behav Anal*. 2014;47(1):151-4.

36. Brinsden H, Lobstein T. Comparison of nutrient profiling schemes for restricting the marketing of food and drink to children. *Pediatr Obes*. 2013;8(4):325-37.

37. Committee of Advertisting Practice (CAP). *CAP Consultation: food and soft drink advertising to children*. 2016. https://www.asa.org.uk/asset/98337008-FA03-481B-92392CB3487720A8/ (accessed 30 Nov 2016).

38. Piacentini M, MacEadyen L, Eadie D. Corporate social responsibility in food retailing. *Int J Retail Distrib Manag*. 2000;28(11):459-69.

39. Sustain. *Press for change at the till: Get the junk off the checkouts!* 2014. https://www.sustainweb.org/childrensfoodcampaign/chuck_junk/ (accessed 31 Oct 2016).

40. Parent's Voice. *Healthy checkouts*. 2014. http://parentsvoice.org.au/our-work/healthy-checkouts/ (accessed 25 Oct 2016).

41. Kerr J, Sallis JF, Bromby E, Glanz K. Assessing reliability and validity of the GroPromo audit tool for evaluation of grocery store marketing andpromotional environments. *J Nutr Educ Behav*. 2012;44(6):597-603.


For peer review only
